# Preparation and Characterization of Naringenin-Loaded Elastic Liposomes for Topical Application

**DOI:** 10.1371/journal.pone.0131026

**Published:** 2015-07-09

**Authors:** Ming-Jun Tsai, Yaw-Bin Huang, Jhih-Wun Fang, Yaw-Syan Fu, Pao-Chu Wu

**Affiliations:** 1 Department of Neurology, China Medical University Hospital, Taichung, Taiwan, ROC; 2 School of Medicine, Medical College, China Medical University, Taichung, Taiwan, ROC; 3 School of Pharmacy, Kaohsiung Medical University, Kaohsiung, Taiwan, ROC; 4 Department of Biomedical Science and Environmental Biology, Kaohsiung Medical University, Kaohsiung, Taiwan, ROC; The University of Tennessee Health Science Center, UNITED STATES

## Abstract

Excessive production of radical oxygen species in skin is a contributor to a variety of skin pathologies. Naringenin is a potent antioxidant. The purpose of the present study was to develop elastic liposomes for naringenin topical application. Naringenin-loaded elastic liposomes containing different amounts of Tween 80 and cholesterol were prepared. The physicochemical properties including vesicle size, surface charge, encapsulation efficiency, and permeability capacity were determined to evaluate the effect of components. The stability of formulation and skin irritation caused by drug-loaded elastic liposomes were also evaluated for assessment of the clinical utility of elastic liposomes. Saturated aqueous solution of naringenin and naringenin dissolved in 10% Tween 80 solution (5 mg/mL) were used as the control group. The result showed that in using elastic liposomes as carrier, the deposition amounts in the skin of naringenin were significantly increased about 7.3~11.8-fold and 1.2~1.9-fold respectively, when compared with the saturated aqueous solution and Tween 80 solution-treated groups. The level of drug was more than 98.89±3.90% after 3 months of storage at 4℃. In a skin irritation test, the result showed experimental formulation exhibit considerably less irritating than the positive control (paraformaldehyde-treated) group, suggesting its potential therapeutic application.

## Introduction

Reactive oxygen species including hydrogen peroxide, superoxide anion, and singlet oxygen are generated as by-products of cellular metabolism primarily in mitochondria, and play a predominant role in many pathological conditions, including immune suppression, photo-carcinogenesis, and photo-aging [1–4]. Excessive generation of reactive oxygen species in the skin is a major contributor for various cutaneous pathologies [5]. Using antioxidants to prevent oxidative skin damage appears to be a promising approach. Naringenin (5,7-dihydroxy-2-(4-hydroxyphenyl)chroman-4-one, C_15_H_12_O_5_, MW 272.3) a flavanone found in many citrus fruits, has been proven to possess anti-inflammatory, antioxidant, and free radical scavenger properties [6,7]. Furthermore, previous studies [8,9] reported that naringenin can increase the tyrosinase activity and melanin content, demonstrating naringenin can be used to prevent oxidative skin damage. Nevertheless, naringenin is a poor water-soluble compound and has minimal oral bioavailability (approximately 5.8%) owing to its largely hydrophobic ring structure [10–12]. Therefore, the purpose of this study was to design a naringenin formulation for topical administration.

In recent years, nano-scale structures such as microemulsions, ethosomes, liposomes and solid lipid nanoparticles have attracted increasing attention because they can provide a better chance for adhesion to biological membranes while delivering therapeutic drugs in a controlled manner. Moreover, nano-scale structures are capable of increased drug loading, sustained release, and the promise of tissue-specific targeting [13–22]. Liposomes are microscopic vesicles with an aqueous core surrounded by one or more outer shell(s) composed of phospholipids in a bilayer. They can incorporate a variety of hydrophilic and hydrophobic drugs, improve the accumulation of the drug at the administration site, and reduce side effects [23–26]. Hence, liposomes have been widely used as safe and effective drug vehicles in topical treatment of disease [27–30]. Modified liposomes such as elastic liposomes were first described by Cevc and Blume [31]. They consist of phospholipids and a single chain surfactant such as deoxycholate, sodium cholate, Tween 80 or Span 80, which can destabilize the lipid bilayers and provide greater flexibility compared to the liposome itself [32–34]. Numerous studies have demonstrated that elastic liposomes could provide potentially deeper permeation of drugs compared to conventional liposomes [15,35,36]. Thus, the present work was aimed at the development of an effective elastic liposome for naringenin topical application. With this purpose, different elastic liposome formulations were prepared. The vesicle size, surface charge and encapsulation efficiency were determined. The permeation properties of drug from these delivery systems through rat-excised skin were evaluated and compared with those of a saturated drug aqueous solution. The stability of formulation and skin irritation caused by drug-loaded elastic liposomes were also evaluated for assessing the clinical utility of elastic liposomes.

## Materials and Methods

### Materials

Naringenin and hesperetin were purchased from Tokyo Chemical Industry (Tokyo, Japan). Polyoxyethylene sorbitan monooleate (Tween 80) and propylene glycol (PG) was from J. T. Baker (Phillipsburg, USA). Epikuron-200 (containing more than 92% of phosphatidylcholine and others of lysoPC, phosphatidic acids, and triglycerides.) was acquired from Cargill, Inc. (Minnetonka, Minnesota, U.S.). Cholesterol and paraformaldehyde were purchased from Sigma-Aldrich (St. Louis, Missouri, USA). All other chemicals and solvents were of analytical reagent grade.

### Naringenin-loaded elastic liposomes preparation

In order to easily evaluate the effect of components, a two-factor three-level factorial design [37] was used to prepare different naringenin-loaded elastic liposome. Each formulation (4 mL) contained 20 mg naringenin and 340 mg other ingredients of cholesterol of 5~15%, Tween 80 of 10~20% and Epikuron-200. Cholesterol and Tween 80 were set as formulation factors. The compositions of all different elastic liposome are listed in Table 1.

**Table 1:** The composition and physicochemical characteristics of naringenin-loaded elastic liposomes.

	Cholesterol		Tween 80		Size	PI	Zeta	EE
	code	mg	code	mg	(nm)		mV	%
F1	+1	52	+1	68	155.0±1.9	0.16±0.03	-16.1±0.7	9942
F2	+1	52	0	52	177.7±3.5	0.19±0.05	-13.4±0.3	99.43
F3	+1	52	-1	34	ND-			
F4	0	34	+1	68	123.7±1.8	0.16±0.02	-11.0±3.7	99.52
F5	0	34	0	52	138.3±1.7	0.13±0.03	-12.3±1.2	99.56
F6	0	34	-1	34	176.1±1.8	0.22±0.02	-15.6±1.5	99.53
F7	-1	17	+1	68	ND-			
F8	-1	17	0	52	147.3±2.9	0.11±0.03	-8.5±1.5	99.59
F9	-1	17	-1	34	132.3±1.3	0.17±0.02	-2.2±2.4	99.64

Each liposome formulation contained 20 mg naringenin and 340 mg of other ingredients of epikuron, cholesterol and Tween 80 in 4 mL aqueous phase.

PI: polydispersity index; EE: Encapsulation efficiency.

ND: no data because the size was over detected level.

The accurately weighed amounts of naringenin, epikuron, Tween 80 and cholesterol were placed in a round-bottom flask, and the mixture was dissolved in 4 mL mixture solvent of chloroform-methanol at a ratio of 1: 1. The organic solvent was removed by rotary evaporation under reduced pressure at 65°C, and then the solvent traces were removed by maintaining the lipid film under a vacuum overnight. The deposited lipid film was hydrated with 4 mL aqueous solution by a probe-type sonicator (UP50H,Hielscher Ultrasonics, Teltow, Germany) for 10 min at 50 W [34]. The final concentration of drug-loaded liposomes was 5 mg/mL.

### Physicochemical characterizes of elastic liposomes determination

The mean vesicle size and zeta potential (surface charge) of the naringenin-loaded elastic liposomes were measured using Zetasizer 3000HSA (Malvern Instruments, Malvern, UK) with a helium-neon laser with a wavelength of 633 nm at room temperature. A 1:80 dilution of the drug-loaded liposome was made using double-distilled water for the measurements. The size values were given as a volume distribution. Analysis time was kept at 60 sec, and average size and zeta potential of the vesicles were determined.

### Encapsulation efficiency determination

The entrapment percentage of naringenin loaded in elastic liposomes was determined by an ultracentrifugation method. The sample was centrifuged at 120,000 rpm for 1 h at 4°C in a Hitachi CS150GXL ultracentrifuge (Tokyo, Japan) to separate the incorporated drug from the free form. There were no vesicles in the supernatant after Zetasizer examination. The supernatant was analyzed by high-performance liquid chromatography (HPLC) [38] to determine the drug encapsulation percentage of the total naringenin load. The percentage encapsulation efficiency of naringenin in elastic liposome was calculated as: Each experimental was performed in triplicate, and the data reported is the mean value.

$$\text{Encapsulation efficiency(\%)}=\frac{\text{Drug content}}{\text{Total drug loaded}}\times 100\%$$

### Skin permeation and drug deposition studies

The experimental protocol was approved by the Institutional Animal Care and Use Committee of Kaohsiung Medical University (Kaohsiung, Taiwan). The Committee confirmed that the permeation experiment followed the guidelines as set forth by the Guide for Laboratory Fact lines and Care. The *in vitro* skin permeation and skin deposition studies of naringenin-loaded elastic liposomes (5 mg/ mL) and control group (the saturated aqueous solution of drug and drug dissolved in 10% Tween 80 of 5 mg/ mL) were conducted by using a modified transdermal Franz diffusion cell. The effective diffusion area of the diffusion cell and receptor cell volume were 3.46 cm^2^ and 20 mL respectively. The abdominal skin was excised from a Sprague-Dawley rat weighing 275–300 g, and then mounted on the receptor chamber with the stratum corneum side facing upward to the donor chamber. Samples of 1 mL were placed in the donor chamber and occluded by parafilm. Twenty mL of pH 7.4 phosphate buffer saline containing 40% PG (drug solubility of 941.50 ±3.54 mg/mL) was placed in the receiver chamber. The temperature of receiver medium was maintained at 37±0.5°C by thermostatic pump and was constantly stirred at 600 rpm by a magnetic stirrer during the experiment. At determined intervals, *i*.*e*., 1, 2, 3, 4, 6, 8, 10, and 24 h, one milliliter of receptor medium was withdrawn via the sampling port and was quantified for naringenin level by a modified HPLC method [38].

At the end of the skin permeation experiments, the donor phase was removed and the rat skin was washed with deionized water to remove the residual naringenin on the skin surface. Then, the skin was dried with cotton wool and the stratum corneum (SC) was removed from the rest of the skin by tape-stripping the skin with 11 adhesive cellophane tapes (Scotch Book Tape no. 845, 3M, St Paul, MN) [39,40]. The first tape was discarded. The other stripping tapes were put in glass tubes containing 2.0 mL of methanol and then shaken horizontally for 1 h. The solution was filtered through a 0.45 mm membrane (Sartorius, Goettingen, Germany). Naringenin in filtrate was determined by HPLC. The epidermis was separated from the dermis with heat application at 80°C for 3 min and the help of forceps [41]. Then, the epidermis and dermis were separately cut into small pieces to extract the drug content present in the skin with methanol. The resulting solution was centrifuged for 10 min at 8533 g, and filtered through a 0.45 mm membrane. The filtrate was analyzed for naringenin by HPLC.

### Chromatographic condition

Hitachi L-7100 series HPLC system and a LiChroCART RP-18e column (125×4 mm I.D., particle size 5 μm) were used in this study. The detection wave was set at 281 nm. The mobile phase of 0.5% triethylamine (adjusted to pH 3.0 by acetic acid) containing 28% acetonitrile was delivered at a rate of 1.0 mL/min. Hesperetin of 200 μg/mL was used as internal standard. The method was successfully validated with coefficient of variation (CV, %) of 3.72%, relative error (RE, %) of 7.57% and a determination coefficient (r) of 0.9998. The limit of quantitation was 0.1 μg/mL.

### Skin irritation determination

The experimental protocol was approved by the Institutional Animal Care and Use Committee of Kaohsiung Medical University (Kaohsiung, Taiwan). The committee confirmed that the permeation experiment followed the guidelines as set forth by the Guide for Laboratory Fact Lines and Care. The hair on the abdomen of the rat was shaved before the skin was randomly divided into three study groups. A glass ring with 2.54 cm^2^ was adhered to the abdomen skin. The experimental naringenin-loaded elastic liposome (F4), aqueous water (normal control group) and 0.8% paraformaldehyde (positive control group) of 0.5 mL were loaded into the glass ring and left for 24 h [28,42]. Then, the tested skin regions were excised and fixed in 4% buffered formaldehyde solution for 24 h. The skins were then embedded in paraffin and sliced transversely. The sections were rehydrated stepwise, stained with hematoxylin and eosin, and observed using an optical microscope (Nikon Eclipse Ci, Tokyo, Japan).

### Stability determination

The naringenin-loaded elastic liposome was stored in dark-brown bottles for protection from light. The stability of drug-loaded liposome formulation (F4) was evaluated via physicochemical properties and drug content at 4°C. The physical stability was evaluated by mean vesicle size and zeta potential measurement over a three-month period.

### Data analysis

All experimental measurements were performed in triplicate. Result values were expressed as the mean value ±standard deviation. Statistical analysis of differences between the experimental formulations was performed using ANOVA test provided by Winks SDA 6.0 software (Texasoft, Cedar Hill, TX, USA). The post hoc Newman—Keuls test was used to check individual differences between groups. A 0.05 level of probability (p < 0.05) was taken as the level of significance.

## Results and Discussion

Typically, liposomes are composed of neutral phospholipids, which are biocompatible molecules. Cholesterol is often added to improve mechanical stability of the bilayer and decrease leakage of the encapsulated materials. Cholesterol has been shown to increase mechanical strength of membrane, affect its elasticity, and increase the packing density of lipid via the “ordering and condensing” effect [43–46]. Some studies have indicated that the cholesterol content might be the crucial factor for the effective delivery of liposome-entrapped drugs into the skin [47,48].

Conventional liposomes are reported to remain confined to the upper layer of the SC and to accumulate in the skin appendages, with minimal penetration to deeper tissues, due to their large vesicle size and lower flexibility of membrane [14,49–51]. Tween 80 is a single chain surfactant. It can act as an “edge activator” and destabilize the lipid bilayers of traditional liposomes, and this then provides greater flexibility of membrane [36,52,53]. However, liposomes in the present high concentration of Tween 80 are unstable; hence, the effect of concentration of cholesterol and Tween 80 on the physicochemical characteristic and skin permeation capacity of liposome was investigated in this study. Naringenin-loaded elastic liposomes containing different amounts of cholesterol and Tween 80 were prepared.

### Vesicle size, zeta potential and encapsulation efficiency

The average vesicle size, zeta potential (surface charge) and encapsulation efficiency of experimental formulations are listed in Table 1. Except for formulations F3 (cholesterol was at a high level and Tween 80 was at a low level) and F7 (cholesterol at low levels and Tween 80 at high levels), the average vesicle size of all formulations ranged from 123.7 to 177.7 nm. The polydispersity index values of the elastic liposomes were obtained in a range of 0.11–0.22, showing homogenous size distribution in all formulations. From Table 1, it can be found that the average size of elastic liposomes tended to become large when formulated with higher levels of cholesterol. On the contrary, the average size tended to diminish, when formulated with higher levels of Tween 80. A possible explanation may be that the edge activator, Tween 80, destabilizes the lipid bilayers of liposomes, thus resulting in smaller vesicles [14]. However, the smallest size was obtained at elastic liposomes containing a medium level of cholesterol and a high level of Tween 80.

The surface charges of experimental formulations ranged from -2.2 to -16.1 mV. The drug-loaded elastic liposomes had lower surface charge when a high level of cholesterol was incorporated. The results agreed with a previous study, which reported that increasing the level of cholesterol in a phospholipid membrane decreases surface charge in the physiological environment [54].

Numerous studies have reported that the elastic liposome could significantly increase solubility of hydrophobic and hydrophilic compounds [15,55]. Naringenin is a poor water-soluble compound; its solubility in water was 41.76 ±0.51 μg/mL. In this study, 5 mg/mL of drug was loaded into the elastic liposomes. The encapsulation efficiency of all experimental formulations was larger than 99%, indicating that elastic liposomes should be a good carrier for naringenin.

### Skin permeation and drug deposition

**Fig 1:**
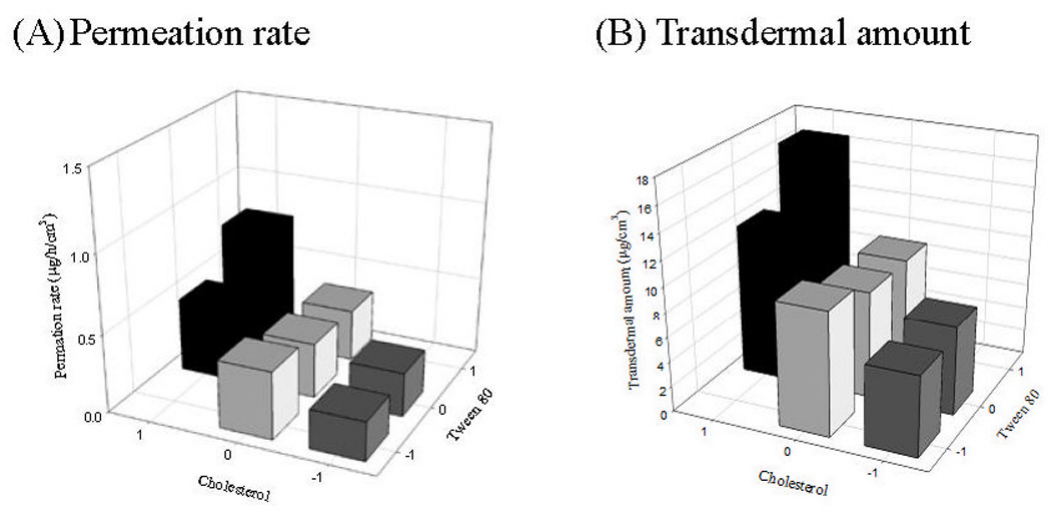
The permeation rate and transdermal amount at 24 h of naringenin-loaded elastic liposomes through rat skin.

**Fig 2:**
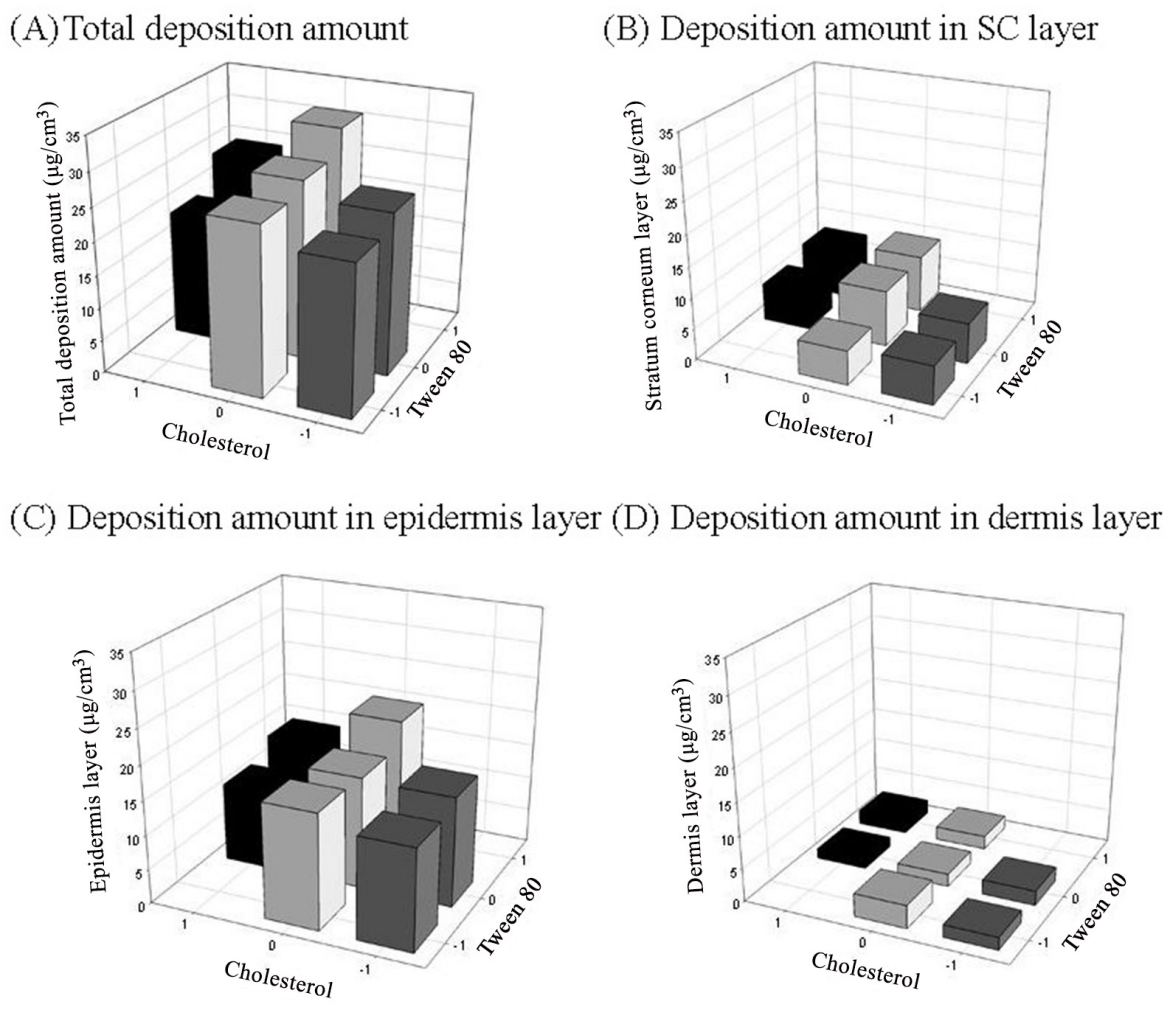
The skin deposition amount of naringenin-loaded elastic liposomes after 24 h treatment.

The cumulative amount transported through rat skin was plotted as a function of time, and the linear regression analysis was used to determine the permeation rate (flux) and permeation mechanism of drug. The result showed that the permeation profiles followed a zero-order model (R^2^ > 0.9915). The permeation rate, cumulative amount at 24 h and deposition amount in three skin layers including SC, epidermis and dermis layer after 24 h treated with naringenin-loaded elastic liposomes with different levels of cholesterol and Tween 80 are presented in Figs 1 and 2. The saturated aqueous solution and 5 mg/mL of drug dissolved in 10% Tween 80 solution were used as control groups to evaluate the enhancement effect of formulations. The permeation rate and cumulative amounts at 24 h were 0.25±0.1 μg/h/cm^3^ and 4.8±2.6 μg/cm^3^ for saturated aqueous solution, 0.37±0.15 μg/h/cm^3^ and 14.4±3.1 μg/cm^3^ for 10% Tween 80 solution, and 0.25±0.05~0.76±0.21 μg/h/cm^3^ and 6.4±0.8 ~16.5±3.4 μg/cm^3^ for elastic liposomes (Fig 1A and 1B). The permeation rate and cumulative amount were increased 1.5-fold and 3.0-fold by using permeation enhancer (Tween 80), indicating Tween 80 was an effective penetration enhancer (p<0.05) [56,57]. When elastic liposomes were used as carrier vehicles, the enhancement ratios were 1.0~3.0-fold for permeation rate and 1.3~3.5-fold for cumulative amount. The result showed that the composition proportions of elastic liposomes would affect the enhancement degree of drug transportation through skin. An appropriate composition proportion of formulation of F1 with high-level cholesterol and Tween 80 could obtain the highest enhancement effect. Its enhancement effect was similar to that of 10% Tween 80 solution. The result agreed with previous studies, which reported that liposome-like vesicles and/or penetration enhancer-containing vesicles could improve the transportation through skin of the drug [27–29].

The deposition amounts of drug in SC, epidermis, dermis layers and total deposition amounts were 0.5±0.1, 1.8±0.5, 0.3± 0.1, and 2.6± 0.7 μg/cm^3^ for the saturated aqueous solution-treated group and 4.4±1.2, 9.3±2.7, 2.2±1.6, and 15.9±2.5 μg/cm^3^ for the 10% Tween 80 solution-treated group. The result showed that naringenin was deposited in different skin layers, particularly in the epidermis layer. Using elastic liposomes as carrier also showed similar results (Fig 2). Hence, the value of total deposition amount was used to evaluate the efficacy of formulations. In comparison with the two control groups, the total deposition amount increased about 6.1-fold when 10% of Tween 80 was used as permeation enhancer (p<0.05) [56,57]. When elastic liposomes were used as drug carrier vehicles, the total deposition amounts (19.0±3.7 ~30.7±13. μg/cm^3^) further increased 1.2~1.9-fold compared with the Tween 80-treated group, indicating that the elastic liposomes had even more potential for naringenin skin transportation (p<0.05). Furthermore, the enhancement efficiency of these elastic liposomes on the deposition amount was more than that on the permeation amount, indicating that the present liposomes were more suitable for topical skin target of naringenin. This finding is in agreement with published studies that reported use of liposomes as carrier may produce higher drug concentrations in the skin layers and lower systemic concentration [14,15,36,58]. The enhancement mechanism of skin delivery includes but is not limited to: the small size of vesicles; the cell membrane-like structure of the vesicles having good biocompatibility; formation of drug reservoirs in the skin; specific liposome-skin interactions; and the elastic vesicles being able to squeeze through intercellular regions of the SC under the influence of the transepidermal water-activity gradient. [14,51,59].

In evaluation of the effect of physicochemical characteristics and compositions of liposomes on the permeation parameters, a non-significant relationship was found between the average size *vs* permeation rate and cumulative amount (*p>*0.05). A possible explanation may be that elastic liposomes being flexible should be able to more easily squeeze through the skin without being affected in size. On the contrary, the total deposition amount in skin increased by increasing the amount of Tween 80 (Fig 2). The result is consistent with previous studies [14,55,58,60], which reported surfactant “Tween 80” can be inserted into the lipid bilayers of liposome thereby creating a “softened” and “flexible” bilayer membrane, thus facilitating elastic liposome rapid distribution into the skin retaining the drug on, in, and below the skin barrier. In cases of elastic liposomes with different added amounts of cholesterol, it was found that the formulation with medium level(s) of cholesterol showed highest skin deposition. It is possible that excess cholesterol resulted in a rigid membrane, and insufficient cholesterol resulted in a looser membrane, both making less-permeable conditions [46,47]. However, the naringenin-loaded elastic liposome with high-level Tween 80 and medium-level cholesterol showed highest total deposition of 30.7±13.7 μg/cm^3^, which was 11.8-times that of saturated aqueous solution.

### Skin irritation

**Fig 3:**
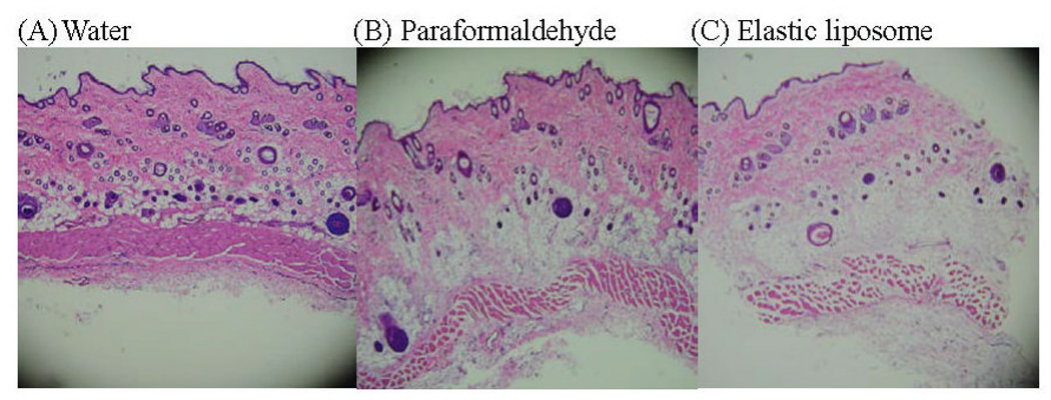
The microstructure of a rat abdominal skin section, viewed under a light microscope.

Formulations might elicit primary skin irritation. Rat skin irritation experiments were conducted to assess the potential irritant effects of the developed elastic liposome formulation. The 0.8% v/v aqueous solution of paraformaldehyde was used as a standard irritant [28,42]. As shown in Fig 3B, a slight edema exfoliation of the stratum corneum, and collagen dissociate was caused by application with paraformaldehyde. Non-significant edema and erythema was found in tested elastic liposome (Fig 3C) and aqueous solution control (Fig 3A), when compared to the positive control group, indicating that the experimental elastic liposome formulation appeared to be safe for transdermal delivery.

### Stability

After three months of storage at 4°C, non-aggregation and creaming were observed. The size and zeta potential of drug-loaded liposome had slight change from 123.7 to 128.1 nm and -11.0 to 12.7 mV respectively, showing non-significant difference. The residual drug content of tested drug-loaded elastic liposome was 98.89±3.90%, indicating that the formulation was stable.

## Conclusions

The naringenin deposition amounts in SC, epidermis, dermis and total skin were significantly increased by using elastic liposomes as drug carrier when compared to the saturated aqueous solution and Tween 80-treated groups. The added contents of cholesterol and Tween 80 showed significant influence on the physicochemical properties and permeation capacity of naringenin from elastic liposomes. The naringenin-loaded elastic liposome with high-level Tween 80 and medium-level cholesterol showed highest deposition of the drug in skin, which was 11.8-times that of the saturated aqueous solution. It has also been demonstrated that naringenin-loaded elastic liposome was stable after three months of storage and produced less skin irritation than that of the standard irritant group. The result suggests that elastic liposome is a promising carrier for naringenin topical application.
